# ‘Doing the hard yards’: carer and provider focus group perspectives of accessing Aboriginal childhood disability services

**DOI:** 10.1186/1472-6963-13-326

**Published:** 2013-08-19

**Authors:** Michelle DiGiacomo, Patricia Delaney, Penelope Abbott, Patricia M Davidson, Joanne Delaney, Frank Vincent

**Affiliations:** 1 Centre for Cardiovascular and Chronic Care, Faculty of Health, University of Technology Sydney, Broadway, P.O. Box 123, Broadway, Australia 2007; 2 Aboriginal Medical Service Western Sydney, P.O. Box 3160, Mt Druitt, Australia 2770; 3 University of Western Sydney, Locked Bag 1797, Penrith, Australia 2751

**Keywords:** Childhood disability, Aboriginal and Torres Strait Islander peoples, Early intervention, Focus groups

## Abstract

**Background:**

Despite a high prevalence of disability, Aboriginal Australians access disability services in Australia less than non-Aboriginal Australians with a disability. The needs of Aboriginal children with disability are particularly poorly understood. They can endure long delays in treatment which can impact adversely on development. This study sought to ascertain the factors involved in accessing services and support for Aboriginal children with a disability.

**Methods:**

Using the focus group method, two community forums, one for health and service providers and one for carers of Aboriginal children with a disability, were held at an Aboriginal Community Controlled Health Service (ACCHS) in the Sydney, metropolitan area of New South Wales, Australia. Framework analysis was applied to qualitative data to elucidate key issues relevant to the dimensions of access framework. Independent coding consistency checks were performed and consensus of analysis verified by the entire research team, several of whom represented the local Aboriginal community.

**Results:**

Seventeen health and social service providers representing local area government and non-government-funded health and social service organisations and five carers participated in two separate forums between September and October 2011. Lack of awareness of services and inadequate availability were prominent concerns in both groups despite geographic proximity to a major metropolitan area with significant health infrastructure. Carers noted racism, insufficient or non-existent services, and the need for an enhanced role of ACCHSs and AHWs in disability support services. Providers highlighted logistical barriers and cultural and historical issues that impacted on the effectiveness of mainstream services for Aboriginal people.

**Conclusions:**

Despite dedicated disability services in an urban community, geographic proximity does not mitigate lack of awareness and availability of support. This paper has enumerated a number of considerations to address provision of disability services in an urban Australian Aboriginal community including building expertise and specialist capacity within Aboriginal Health Worker positions and services.

Increasing awareness of services, facilitating linkages and referrals, eliminating complexities to accessing support, and working with families and Aboriginal community organisations within a framework of resilience and empowerment to ensure a relevant and acceptable model are necessary steps to improving support and care for Aboriginal children with a disability.

## Background

Aboriginal and Torres Strait Islander (hereafter, Aboriginal Australian) children experience inferior health outcomes when compared to other Australian children and this includes higher rates of disability [1,2]. The issues around Aboriginal childhood disability are complex and multifaceted. The first years of a child’s life are critical periods for social and emotional development as well as health [3,4]. The antecedents of disability can be associated with environmental factors rather than biological ones. Aboriginal Australians are twice as likely to engage in or be exposed to risk factors that can lead to disability, such as smoking, binge drinking, obesity, using illicit drugs, poor housing conditions and being victims of violence [5,6]. Disability, compounded by social disadvantage, can lead to a perpetuating cycle of health disparities and inequities. As a consequence, Aboriginal Australians with a disability have been described as ‘doubly disadvantaged’ [7]. Given that Aboriginal Australians experience high levels of disadvantage, if ignored or undertreated, disability at an early age may result in a greater likelihood of unemployment, lower socioeconomic status and interaction with the juvenile justice system [3,8].

Little is known about the prevalence and experience of those with a disability within the Aboriginal population, particularly children and those living in urban areas [9]. It was not until 2002 that a national survey of Aboriginal disability was undertaken [5] and not until 2006 that a question was included on a national survey that asked specifically about disability in Aboriginal children aged 15 and under [2]. While the available data are limited, the 2006 survey highlighted that Aboriginal children are more likely to experience disability than other Australian children. Aboriginal children aged 0–4 years were more likely to have a need for assistance with self-care, mobility or communication than non-Aboriginal children of the same age (ratios of 1.2 for males and 1.3 for females) [2]. For those aged 5–18, Aboriginal males and females were 1.3 and 1.5 times as likely to have a profound or severe disability as their non-Indigenous counterparts [2]. In Western Australia, an estimated 2% of Aboriginal children aged 4–17 were reported by parents and carers to need help with basic activities associated with daily living such as eating, dressing, bathing and going to the toilet [2].

The disproportionately high prevalence of middle ear disease and its complications in Aboriginal children can lead to disabling chronic ear disease, hearing impairment and negatively affect education, social circumstances and quality of life [10,11]. In 2004–05, rates of middle ear disease were four times as high among Aboriginal children aged 0–14 years as non-Aboriginal children in the same age group [3]. Five per cent of Aboriginal children are reported to have complete or partial deafness, compared to 1% of non-Indigenous Australians [12], yet they are less likely to wear hearing aids [13].

Although Aboriginal Australians are more likely to have a disability than non-Indigenous Australians, they are less likely to use services [14-16]. The Aboriginal Disability Network of New South Wales has highlighted that there are very few Aboriginal people with disability who have their needs met in a meaningful way [6]. Aboriginal Australians face significant barriers to accessing disability support services. Social marginalisation, mistrust in government services and agencies, cultural attitudes towards disability, a lack of facilities that provide culturally appropriate services, remoteness, child care, and transportation have been reported as barriers to accessing services [5,6]. Penchansky and Thomas described healthcare access as being comprised of five dimensions; availability, affordability, accessibility, accommodation, and acceptability [17]. Each of these dimensions plays a critical role in determining access to health care services. Availability depicts the volume and range of health resources; affordability refers to costs of services; accessibility refers to the location of services; accommodation involves whether the organisation of health care fits the users’ demands; and acceptability refers to whether the health service fits with the users’ attitudes and characteristics [17].

In order to meaningfully address this problem, it is important to describe contextual, cultural and service-related factors which may impact on access. In spite of a range of factors across the sector, it is important to recognize the heterogeneity of Aboriginal communities and the importance of considering every community differently [18]. This study sought to ascertain the factors involved in accessing services and support for Aboriginal children with a disability in an urban setting.

## Methods

Community forums using focus group methods [19] were undertaken to ascertain perspectives of services and support for Aboriginal children with disability. Aboriginal people have experienced decades of colonial oppression and as a consequence, it is important to consider methodological approaches. Based upon consultation with community leaders it was considered that discussions in non-threatening and culturally appropriate settings would be most appropriate [20]. The focus group method of group discussion was considered advantageous because of the narrative method favoured in Indigenous research and the opportunity to grasp collective experiences and interpret these collectively [21]. Moreover, diffusing views and opinions in a collective forum can have high utility and data clarity, particularly in health services research [22]. In an effort to obtain multiple perspectives, two separate forum were held; one for service providers and one for carers of young Aboriginal children with a disability. The carers were family members or parents of the children, however for the purposes of preserving anonymity, participants will be described as carers. Similarly, we have minimised the potential reporting of identifiable contextual and socio-demographic information. Cultural supervision and mentorship of the project was undertaken by a community leader. Participants provided consent and were aware that the information would be published with the aim of improving service delivery. Ethics approval to undertake this study was granted by the Aboriginal Health and Medical Research Council (762/10) and this article was approved for publication by the governing Board of the Aboriginal Community Controlled Health Service (ACCHS).

### Recruitment

Each forum was advertised via flyers posted in an ACCHS, emailed to relevant professional groups and disability networks within three proximal health district areas, or given directly to community members by Aboriginal Health Workers (AHWs) or ACCHS staff. Recruitment adopted an inclusive approach whereby the definition of disability remained broad, encapsulating physical as well as intellectual disability or sensory impairment. Individuals who agreed to participate were contacted on the day prior to forums to confirm attendance.

### Setting

This study took place in an outer metropolitan area of Sydney, Australia. The region has entrenched socio-economic disadvantage with high rates of public housing and people living below the poverty line [23]. The area has a large proportion of pre-school aged children (less than 5 years old), and Aboriginal people represent 1.6% of the population. The local health district provides health services to the surrounding area comprising 833,000 residents and includes five hospitals, three of which are large teaching hospitals and one is a children’s hospital, and seven community health facilities. Primary care within the region is provided largely by general practitioners in private practice. There is one large Aboriginal community controlled health service which provides multidisciplinary primary health care to the Aboriginal community of western Sydney. A range of governmental and non-governmental disability services are available within the region.

The group discussions took place in a community controlled facility deemed a culturally appropriate and familiar community venue [20,24]. Transportation was provided, as needed, to facilitate participation and carers were advised that their children were welcome. Issues such as transportation and childcare have been identified in previous studies as barriers to participating in health programs [25].

### Procedure

The carer forum was held on a weekday in a private meeting room and was moderated by one Aboriginal researcher and one non-Aboriginal researcher. Questions and discussion centred on carers’ perspectives and experiences, barriers to accessing support, and needs and preferences for support.

The provider forum was held on a different day with all research team members in attendance. One member of the project team facilitated discussion and three team members scribed. Questions and discussion centred on providers’ perspectives and experiences of barriers to obtaining disability support, both in their direct professional capacity and what they have witnessed as impacting carers/families. Proposed solutions were also discussed with participants by exploring noted barriers and potential solutions that emerged from the group discussion. The moderator provided opportunity for individuals’ views and opinions to be discussed, drawing parallels and differences from a range of opinions. Three scribes (non-Aboriginal researchers) took notes throughout the meetings which were not audio recorded to facilitate a safe and non-threatening environment. Key points were summarised at the end of each focus group and the participants were asked to confirm accuracy.

### Data analysis

Handwritten notes included participants’ quotes and paraphrased excerpts from proceedings. Notes were typed and any potentially identifying information was removed prior to applying framework analysis [26]. Analysis began with close reading of the raw data and note-taking to facilitate immersion. Key issues were identified and noted and then considered within the dimensions of access framework (availability, affordability, accessibility, accommodation, and acceptability) [17]. All textual data was systematically indexed according to dimension of access. Any material that did not appear to fit the framework was retained and categorised separately.

Sections of text were charted according to the dimensions of access [17] and participant group and then distilled into summaries of experiences, perspectives, and illustrative quotes. Associations between concepts within the five dimensions led to development of sub-themes and explanations. To ensure trustworthiness of the analysis, another member of the research team performed independent coding consistency checks whereby they were asked to allocate raw data to categories including dimensions of access [27]. Differences were resolved through discussion with a third author. Upon consensus, final analysis was reviewed by the project steering committee. Importantly, the local community was in control of all key points of the data collection and participated in interpretation of findings.

## Results

Five Aboriginal women attended the carer forum which lasted two hours. Although several more carers indicated that they would attend, a community member’s funeral was held at the same time, making attendance at the forum unfeasible for several people. Despite few participants, the depth and length of discussion conveyed an appreciable volume of rich data. Carers’ engaging and articulate discussions centred on topics reflecting several dimensions of access including availability, affordability, accommodation, and acceptability; reflective of their ongoing lived experiences. The carers reported a range of disabilities in their children which included moderate to severe cognitive disability, autism, and syndromal multisystem disability. Participants described experiences of accessing services for a wide range of needs including health care, education, housing, financial and social support, as well as dedicated disability services. Participants began by explaining that in the Aboriginal community, many children with a disability are cared for by someone other than a parent and these arrangements are often informal, which can challenge service access.

Seventeen providers from a range of government and non-government funded disability, health, and social service organisations attended the one hour providers’ forum. Input centred mainly on accessibility, particularly emphasising the complexities of the system. Both groups commented to a lesser extent on the other dimensions of access. Data are presented below according to dimension of access rather than participant group, however Table 1 provides this differentiation.

**Table 1:** Forum data by access dimension

**Dimension of Access**	**Barriers to Access**		**Carers**	**Providers**
**Availability**	**Unmet need for information**	Services, support, diagnoses	Need for information on child’s disability and available support was a prominent concern. Women lacked an understanding of what support existed, even within their local communities. Few had internet access, sought information on disability at a public library. Carers felt they had to educate themselves and that current health education channels were not reaching them adequately. Regarding family access to information, in one instance, a carer described a delay in hearing her child's diagnosis: “*It was months after she was diagnosed that I found out how serious this was.”*	Participants stated that a lack of knowledge of available services was a prominent factor in Aboriginal carers not accessing disability services. People were generally unaware of what services were available and that there was not enough information available to let people know about services. Some service providers, too, were unaware of the breadth of services offered.
		Financial support	Need for information on availability and eligibility for financial support from government welfare organisations currently and in future	
	**Insufficient supply of programs and services**	Treatments delays	Long waitlists for case worker, specialists, and allied health professionals meant time without treatment or therapies, during crucial stages of development when support/assistance is most vital.	Delay between assessment and treatment (eg. waitlists to see speech pathologists ranged from 6 – 24 months)
		Men's programs	No supports/support groups in place for fathers or male carers despite a great need	
		Transitional services, programs for teens	Perceived a significant gap in available services for teenagers and transitional arrangements to assist in adjustment from school to work, work experience, university, or employment	
		After school cultural programs	No culturally appropriate after-school activities available for their children with a disability:	
			*“Where do the kids go that have fallen down the cracks?”*	
		Childcare and school placements; disability support in schools	Difficulty in getting school or child care placements for their children; few schooling options due to the lack of vacancies and lack of appropriate disability support services. Students are placed in classes according to need and the school’s capacity to address this need.	
		Tutoring support	Unable to secure a position in a state school she perceived as meeting her child’s needs, one carer reported needing tutoring support for her child whom she was home schooling, but did not know how to access this assistance.	
**Affordability**	**Out-of-pocket expense**	Respite	Private respite services were accessed by one woman due to inflexible government-subsidised services, but this was expensive and unsustainable.	
		Childcare	The cost of child care, in particular, a place where disability or delay is often identified, impeded use of this service.	
		Tutoring	Cost of tutoring support for child being home schooled is high; carer resigned from job to home school which increased financial stress and social isolation.	
		Transport		Cost of public transport to various agencies and appointments can become expensive.
**Accessibility**	**Pathway complexity**	Confused pathway and processes inhibit access	Given the many obstacles, the complexity of unclear pathways, and denials by health professionals and educators that a problem exists and warrants support, one woman stated: “*Carers have to do the hard yards to get support.”*	Complexity and lack of communication led to significant delays in children receiving services. For instance, it was reported that children sometimes are teenagers before they are linked to support. Participants commented that their case managers found it hard to locate and communicate with other disability services. One participant commented: “*It shouldn’t be this hard.”* They discussed the complexity for health professionals within this system and highlighted the lack of cross-sector communication.
			This was linked to insufficient communication between organisations; information about a child’s condition was not shared for purposes of facilitating treatment or support.	Referral process confusion; referrals initiated by school and councils rather than by families, implicating schools as important entities within the process.
				The absence of a centralised case management body was perceived as problematic.
		Information Technology complexity		Systems to facilitate both provider and carer access of services and networks were perceived as not user-friendly. In particular, a health service provider website and an excess of complicated forms for carers to complete were obstacles to accessing services.
				A dual diagnosis can complicate service access and provision for a carer and the child. The system reportedly was confused and slowed by multiple differing needs for one client.
	**Reputation as inaccessible**	Service reputation as inaccessible a deterrent		Expectation of delayed service (perceived as non-responsive or slow to respond) based on previous experience with disability support organisations led to avoidance of said agencies; perceived crisis-centred only
	**Physical location**	Travel burden	One woman and her family moved from a rural area for better service access, but still faced several months wait for her child's treatment.	Travelling long distances from rural communities while simultaneously caring for other children were cited as barriers to accessing services. In metropolitan areas, travelling on public transport to various agencies and appointments can be time consuming and inconvenient, particularly with small children.
**Accommodation**	**Unmet Communication needs**	Timing, language, and style of health professionals interactions	Pamphlets and other literature on medications did not satisfy the need for information; health professional jargon and emotional response to diagnosis impeded comprehension. One carer’s perspective of hearing the diagnosis: “*Your whole world is crumbling down…”* Need longer contact time with the health professional or other suitable person and more opportunities for understanding this information. They also needed broader referrals and education.	The information on disabilities is hard for Aboriginal families to understand and interpret the signs.
			Parental experience and expertise was not recognised during interactions with mainstream services while attempting to obtain support for her child: “*They don’t listen, they tell us.”*	
		Culturally appropriate communication	Frustration of not being heard; eg. respite provider would not use Aboriginal language with child, despite having been advised that this would enable communication; carer perceived this as an example of Aboriginal language differences not being respected and consequently making it harder for Aboriginal children with a disability to have their needs met.	
	**Administrative Burden**	Excessive paperwork	For some, a barrier to obtaining financial support was the amount of paperwork that needed to be completed and necessary documentation.	
	**Service inflexibility**	Respite restrictions	Government-subsidised respite policies and services were described as restrictive, inflexible, not family-centred, crisis-driven, and not meeting carers’ needs and commitments (eg. one woman mentioned that she was given respite on a weeknight outside of business hours. This timing meant that she was unable to undertake an activity which would make her feel that she had had a break).	“*Disability is a family issue*.”
		School-based support	Need for more tutoring support for mainstream-placed students; without this, children may leave the school system early or require home schooling (eg. unable to secure a position in a state school she perceived as meeting her child’s needs, one carer resigned from her job and began home schooling, which increased her social isolation and financial stress)	
		Diagnostic & geographic rigidity restrict and delay access to services	Difficulty accessing support when developmental problems were not obvious, visible, or usual; one carer explained that due to her child’s non-academic achievements, she had to endure multiple tests to ‘prove’ her disability. Her child’s teacher attributed her difficulties in school to a pre-occupation with extracurricular activities, as repeated by this carer: “*She said, ’if she didn’t spend so much time participating in (extracurricular activities) she would be fine at school.’”*	Disability assessment is highly structured and results in a diagnosis or label; carers may prefer a holistic approach to understanding the child and their circumstances. Rigid criteria for access to services, such as diagnostic assessments and geographic boundaries that did not take into account the individual needs of the family and child creating uncertainty for carers and providers and restricting and delaying access to needed services.
**Acceptability**	**Remnants of colonisation and trauma**	Historical distrust	A deterrent from help-seeking was concern that children might be taken away by government child protection services.	Consideration of historical context of welfare organisations and ‘*white history*’ (referring to white Anglo-Australian cultural dominance and history of colonisation, oppression, and removing of Aboriginal Australian children from their families by government welfare organisations). This may influence carers and service providers to avoid engagement. Group consensus regarding trust as significant barrier that inhibited families accessing disability services, particularly via government organisations.
			Based on their own and others’ experiences, the women believed that Aboriginal people were made to feel as if they had caused their child’s disability; perceived assumptions and generalisations about substance abuse and unemployment: “*They [organisation staff] make you feel like you caused it [the disability].”* This woman explained that neither she nor her husband drink alcohol, smoke, or use drugs, yet she felt strongly that she was being blamed for her child’s disability: “*You could be an angel and they would still criticise you.”*	
		Perceptions of racism and blame	Felt they were under increased scrutiny, had to prove that they needed support, were considered undeserving, or were being judged negatively for needing assistance. Carers perceived that ‘*the system*’ favours or prioritises parents who appear not to be coping or have obvious problems.	
			Another deterrent was the perceived attitude of some organisations: “*They make you feel like they are doing you a favour by giving you respite.”*	
		Disregard for culture	One respite service provider was reported to have failed to communicate in a culturally appropriate way which would have facilitated care of a child having been advised that this would enable communication. She believed this behaviour made it harder for Aboriginal children with a disability to have their needs met.	
		Inappropriate handling, labeling, management		Another barrier to trust was the perception that there were too many people involved in the process. Families that needed support felt that they were being passed around to different people.
				Disability assessment is highly structured and results in a diagnosis or label. This may be off-putting to carers as it does not reflect a holistic approach to understanding the child and his or her context and circumstances.

### Availability

Participants reported an insufficient supply of programs and services with waiting periods extending from months to years for critical time-sensitive therapies. Perceived non-existent programs included support programs for male carers, transitional services for adolescents and young adults, after-school culturally appropriate programs and in- and out-of-school tutoring support. Availability of childcare and supported school placements was inadequate.

Awareness of available services is not explicitly represented within Penchansky and Thomas’s framework, however, it emerged as an important issue for both carers and providers who expressed uncertainty and ignorance regarding support available. Unsure of the implications of diagnoses, lack of readily available information on disability and services available delayed action. Carers felt that current health education channels were not reaching them adequately, so they had to educate themselves. Although support provided by ACCHSs was seen as culturally appropriate and important, it was suggested that dedicated and knowledgeable disability support workers within such organisations would be required to meet people’s needs for health education and service awareness. Few participants had internet access and one participant sought literature from a public library. Unmet needs for information regarding financial support was also reported. Providers also revealed they were not aware of the breadth of services offered in their area.

### Affordability

Although most disability services could be accessed without cost, carers described burdensome expenses that arose given the lack of availability of these services and support. Costs incurred were for childcare, private tutoring, and respite. Providers noted ongoing public transport costs associated with carers bringing children to multiple appointments.

### Accessibility

The dimension of accessibility concerns geographic location and physical premises. Although the forum had a predominantly metropolitan focus, providers described travel burden associated with rural carers attending distant appointments while caring for other children. Living in or re-locating to the metropolitan area often meant time- consuming and inconvenient travel on public transport, particularly with small children.

For the purposes of this dataset, conceptualisation of accessibility extended beyond physical entry and to engagement with the service system. Complexities of the system were prominent in providers’ discussions and the frustration they felt was apparent. Complexity and lack of communication between disability services and different professional sectors led to significant delays in children receiving services. For instance, it was reported that children sometimes are teenagers before they are linked to support. Confusion regarding assessment and therapeutic pathways and referrals, absence of a centralised management system, information technology perceived as ‘not user-friendly’ and incapable of managing multiple diagnoses were also reported. Rigid criteria for access to services, such as diagnostic assessments and geographic boundaries that did not take into account the individual needs of the family and child, was reported as creating uncertainty for carers and providers and as restricting and delaying access to needed services, Individual providers’ perceptions of government service provider organisations as characterized by prolonged waiting periods and only responding to crises led them to circumvent standard pathways in attempts to access more timely care for their clients. They reportedly experienced great difficulty and confusion accessing these services in their daily work, found it hard to locate and communicate with other services, and lacked time to attend meetings and events that might facilitate collaboration and network building.

### Accommodation

Carers discussed several examples of services and providers not meeting their or their children’s needs with respect to their social, educational, and cultural backgrounds. The language, style, and duration of health professional interactions did not always satisfy information or personal needs. Carers preferred to have additional time to discuss and understand information provided and to be given the opportunity for broader education and discussion of referral options. Respite services were described as inflexible, not family-centred, crisis-driven, and not meeting carers’ needs and commitments. The volume of paperwork and documentation required to receive financial assistance represented yet another demand on already overburdened carers.

Providers did not meet children’s needs when they did not incorporate culturally appropriate language and assessed the child according to rigid diagnostic criteria, rather than through holistic assessments that included the sociocultural context of the child. There was an expressed need for more school-based support for mainstream-placed students to avoid early departure of children from the school system.

### Acceptability

Participants considered that Australia’s history of colonisation and oppression of Aboriginal peoples and the forcible removal of children from their families impacted on experiences of Aboriginal families and children and interactions with mainstream services. These experiences and racism have engendered distrust of government organisations and has influenced carers, as well as some service providers, to avoid engagement with disability services. Examples of unacceptable practices included disregard of Aboriginal culture and language, rigid, non-holistic approaches towards disability assessment, and handling by multiple service providers.

Solutions and preferences for services and service access were proposed by both groups (Table 2). Aboriginal Community Controlled Health Service-based initiatives that facilitated education and support and dedicated knowledgeable Aboriginal Child Disability Workers were carers’ preferred models. Providers noted the importance of working within a family-centred, flexible, and centrally managed model. Both school- and ACCHS-based data collection were endorsed to allow informed policy and practice. Increased awareness of disability supports and inter-sectoral partnerships were endorsed.

**Table 2:** Carer and provider-proposed solutions to address barriers to access

**Solution Categories**	**Carer-preferred solutions**	**Provider solutions**
**ACCHS-based support**	Provide education, workshops, information sessions, and support groups (especially for children with intellectual disability) for families	Grassroots-based; educating families and teachers on disability and available services; focus on prevention
	Opportunities for carers to speak with health professionals/service providers face-to-face (rather than computer or phone).	Work within a family-centred, flexible model, personal approach, holistic approach
	Collect accurate data on number of children in the community with disabilities to inform local and government strategies	
**Aboriginal Child Disability Support Workers**	Need for a dedicated support worker/ Aboriginal Child Disability Worker with disability expertise - role in care navigation, health education, interpretation of medical jargon and liaison support with services	Work within a centralised, case manager model; explore other effective and acceptable models (including funding models) in urban Aboriginal populations
**Increased flexibility**	Increased flexibility and responsiveness of respite services	Minimise red-tape and rigid criteria, particularly during crisis; increase flexibility for Aboriginal families
**School-based support**	Utilise school data on Indigenous and disability status to ensure adequate resource distribution; facilitate linkages between other support agencies and schools	Increase awareness of disability and support available across professions and within communities; increase opportunity for network building across sectors
**Contact Information**	Routinely updated listing of services and contacts for support (general and disability- specific information; with consideration for child’s developmental stages)	
**Inter-sectoral partnerships**	Partnerships between schools, parents, and community controlled organisations were also seen as strategies to enable access to services.	

## Discussion

This study has elucidated several barriers to service access experienced by Aboriginal families of children with disabilities. It has demonstrated that although there are dedicated disability services, these are not always available, accessible, accommodating, affordable, or appropriate. Common to both carers and service providers was dissatisfaction around complexity and inflexibility of services.

Although awareness of available services is not explicitly represented within Penchansky and Thomas’s framework, it emerged as an important issue for carers and providers who expressed uncertainty and ignorance regarding support available. A lack of schools’ awareness of available services, such as hearing and developmental screening, has been noted previously [28]. Perhaps awareness of services is assumed within the framework or subsumed within the availability or accessibility dimensions. Indeed, awareness of services and availability were more prominent in the data than concerns over cultural appropriateness; however, it can be argued that awareness of a service must occur prior to attempting to access it and perceive appropriateness. This emphasis underscored that basic promotion of services was grossly inadequate, as perceived by consumers and providers.

Despite the preponderance of literature centring on rural and remote Aboriginal children [29,30], a finding of this study is that geographic proximity does not necessarily facilitate availability. This finding supports a previous report of one in eight Aboriginal Australian children, including those whom are urban-dwelling, waiting longer than recommended for some services [31]. Being located in a major metropolitan area, such as Sydney, is not a guarantee of access. Despite the health infrastructure of the Western Sydney area, our finding of reports of delays and service gaps challenges the myth of urban centres characterized by efficiency and availability. This suggests need for services is a national issue, not just a problem of geographic isolation.

Although availability of services is paramount, services must also meet the needs of Aboriginal families; a mandate that has not always been realised and is often considered a secondary requirement [32]. It can be argued, however, that these dimensions of access are equally important.

The historical context and institutionalized racism and discrimination were discussed at both forums. The legacy of stolen generations and a recent resurgence of government control [33] has contributed to fear and distrust of organisations, marginalisation, disadvantage, and fear that children will be taken [34] making some Aboriginal people reluctant to access support and services [6]. Given that the first years of a child’s life are so critical, it is vital that carers can access services without fear of racism, discrimination, or chastisement. Health providers must also be aware of the stigma around Aboriginal Australian disability; that is, institutionalized attitudes around the causation involving assumptions of drug dependence, alcoholism, and unemployment. Whether implicit or explicit, these issues must be considered and addressed in the provision of disability services within the Indigenous community.

Although there has been a focus on improving access to culturally appropriate services, this has not been as pronounced in the pre-school setting which represents an excellent opportunity for interventions. In Australia, just a quarter of young Aboriginal children attend early childhood services such as pre-school [35]. Developing and evaluating cost effective quality early interventions in pre-school and childcare are key points where development indicators can trigger support, potentially making a difference to life-long outcomes.

This study and others identify that promoting health literacy in Australian Aboriginal people requires greater incorporation and engagement with an Aboriginal worldview [36]. Setting programs in culturally safe and accessible locations, such as an Aboriginal health or educational setting, and involving Aboriginal health workers is of critical importance [20,37,38]. Also important is improved connections between non-Aboriginal and Aboriginal health workers, and Aboriginal and non-Aboriginal services. Mainstream service providers need to work closely with AHWs and ACCHSs as they play an essential role in overcoming cultural and other barriers [39,40]. A model of shared client management should be implemented. Mere involvement of AHWs and Aboriginal services, however, is not enough. Building expertise and capacity within these positions and services is critical, yet this is not currently available in many Aboriginal organisations. Although funding for an Aboriginal Disability Support Worker would be a positive step, it is essential that this position have specialist knowledge of disability and services in the area.

Family-centred solutions were called for, several of which have been previously reported in the literature. Among these are including involving the family in bi-cultural education to facilitate family engagement in primary health prevention and treatment [41]. Building relationships over time, providing opportunities for discussion, and an emphasis on the abilities of the child rather than the disability have been noted [42]. Other important gaps identified were services to support male carers, in- and out-of- school needs of adolescents, and respite services [43]. Table 3 summarises recommendations to improve access.

**Table 3:** Recommendations to improve access according to dimensions of access framework

	
**Availability**	Geographically accessible and acceptable waiting times; the need for Aboriginal Health Workers in navigating the system
**Affordability**	Consideration of additional out of pocket expenses associated with caregiving, medical and educational services
**Accessibility**	Geographically accessible, simplification of cross-sector communication in health as well as social services, child care for siblings, transport support
**Accommodation**	Need for receptive, flexible and responsive assistance that meets the needs of the whole family and proximity to support
**Acceptability**	Culturally appropriate services that recognise the importance of the Aboriginal Community Controlled Sector and recognise a reconciliation agenda

Additional implications for policy include the need for development of clear guidelines regarding the referral processes of Aboriginal children with disabilities and enactment of policies to address financial barriers.

### Strengths and limitations

The small self-selected sample size means that these findings are not necessarily representative of other Aboriginal populations or service providers. However, participants were targeted on the basis of their expertise and lived experience with disability, particularly in within the context of this urban Aboriginal community. Interestingly, the unforeseen funeral that kept several carers from participating, poignantly illustrates both the high mortality rate and solidarity of the community. Participation could be enhanced by including multiple forum events and other consultation opportunities to accommodate the many priorities and responsibilities of community members. The focus group style forums enabled in-depth descriptions of experiences and vocalisation of concerns in a safe atmosphere. The system of health care provision often fails to meet the needs of vulnerable groups, thus, a strength of this study is the opportunity to hear directly from the carers of Aboriginal children as they discussed their needs. Their views can frame a consumer driven, Aboriginal family-centred approach to ameliorate barriers to access.

## Conclusions

Service providers’ experiences of difficulty and confusion accessing disability services in their daily work corroborate the challenges faced by Aboriginal families and reflect a system in need of reform and streamlining. The magnitude and impact of the inadequate care model currently applied to Aboriginal childhood disability signals an urgent need for change.

These findings have implications for not only policy, service planning and provision, but also for primary health care providers, schools and teachers, and front-line personnel within government and non-government organisations. Interdisciplinary and inter-organisational holistic collaborations are needed to support the multi- dimensional needs of families in their quest to ensure their children’s health and well-being.

## Abbreviations

ACCHS: Aboriginal community controlled health service; AHW: Aboriginal health worker.

## Competing interests

The authors declare that they have no competing interests.

## Authors’ contributions

MD contributed to study design, data collection and analysis, and drafting the manuscript. PD contributed to study conceptualisation, data collection and analysis, revising the manuscript, and cultural mentorship. PA contributed to planning the study, data collection and analysis, and revising the manuscript. PMD contributed to planning the study, data collection and analysis, and revising the manuscript. JD contributed to study conceptualisation, review of the manuscript, and cultural mentorship. FV contributed to study conceptualisation, review of the manuscript, and cultural mentorship throughout the study. All authors read and approved the final manuscript.

## Pre-publication history

The pre-publication history for this paper can be accessed here:

http://www.biomedcentral.com/1472-6963/13/326/prepub

## References

[B1] Ou L Chen J Hillman K Eastwood J The comparison of health status and health services utilisation between Indigenous and non-Indigenous infants in Australia Aust New Zeal J Publ Health 2010 34 1 50 56 10.1111/j.1753-6405.2010.00473.x 20920105

[B2] Australian Institute of Health and Welfare (AIHW) Aboriginal and Torres Strait Islander Health Performance Framework 2008 Report, Detailed Analyses 2008 Canberra: AIHW

[B3] Steering Committee for the Review of Government Service Provision Overcoming Indigenous Disadvantage: Key Indicators 2009 2009 Canberra: Productivity Commission

[B4] Maggi S Irwin LJ Siddiqi A Hertzman C The social determinants of early child development: An overview J Paediatr Child Health 2010 46 11 627 635 10.1111/j.1440-1754.2010.01817.x 20796183

[B5] Australian Government Productivity Commission Disability Care and Support Report 54 2011 Canberra: Productivity Commission

[B6] Aboriginal Disability Network New South Wales Telling it like it is: a report on community consultations with Aboriginal people with disability and their associates throughout NSW, 2004–2005 2007 Sydney: Aboriginal Disability Network New South Wales

[B7] Bostock L Access and equity for the doubly disadvantaged Aborig Isl Health Work J 1991 15 4 10 15

[B8] Calma T Preventing crime and promoting rights for Indigenous young people with cognitive disabilities and mental health issues. Edited by Priday E 2008 Australian Human Rights Commission: Sydney

[B9] Comino E Craig P Harris E McDermott D Harris M Henry R Jackson Pulver L Kemp J Knight J The Gudaga Study: establishing an Aboriginal birth cohort in an urban community Aust N Z J Public Health 2010 34 S9 17 20618303 10.1111/j.1753-6405.2010.00546.x

[B10] Grevers G First International Roundtable ENT Meeting Group Challenges in reducing the burden of otitis media disease: an ENT perspective on improving management and prospects for prevention Int J Pediatr Otorhinolaryngol 2010 74 6 572 577 10.1016/j.ijporl.2010.03.049 20409595

[B11] O’Connor TE Perry CF Lannigan FJ Complications of otitis media in Indigenous and non-Indigenous children Med J Aust 2009 191 9 Suppl S60 64 19883359 10.5694/j.1326-5377.2009.tb02929.x

[B12] Australian Bureau of Statistics National Aboriginal and Torres Strait Islander Health Survey Australia 2004–05 ABS cat no 47150 2006 Canberra: ABS

[B13] Strange A Johnson A Ryan B-J Yonovitz A The stigma of wearing hearing aids in an adolescent aboriginal population Aust N Z J Audiol 2008 30 1 19 37 10.1375/audi.30.1.19

[B14] Australian Institute of Health and Welfare The health and welfare of Australia’s Aboriginal and Torres Strait Islander people: an overview 2011 Canberra: Australian Institute of Health and Welfare

[B15] Australian Institute of Health and Welfare Aboriginal and Torres Strait Islander people with disability: Wellbeing, participation and support 2011 Canberra: AIHW

[B16] Australian Institute of Health and Welfare Disability support services 2009-10: report on services provided under the National Disability Agreement Disability series 2011 Canberra: AIHW

[B17] Penchansky R Thomas JW The concept of access: definition and relationship to consumer satisfaction Med Care 1981 2 127 140 7206846 10.1097/00005650-198102000-00001

[B18] Kruske S Belton S Wardaguga M Narjic C Growing up our way: the first year of life in remote aboriginal Australia Qual Health Res 2012 22 6 777 787 10.1177/1049732311432717 22218266

[B19] Acocella I The focus groups in social research: advantages and disadvantages Qual Quant 2012 46 4 1125 1136 10.1007/s11135-011-9600-4

[B20] Abbott P Davison J Moore L Rubinstein R Effective nutrition education for Aboriginal Australians: lessons from a diabetes cooking course J Nutr Educ Behav 2012 44 1 55 59 10.1016/j.jneb.2010.10.006 21782521

[B21] Gorman D Matching research methodology to Australian Indigenous Culture Aborig Isl Health Work J 2009 33 3 4 7

[B22] Halcomb E Gholizadeh L DiGiacomo M Phillips J Davidson P Considerations in undertaking focus group research with culturally and linguistically diverse groups J Clin Nurs 2007 16 6 1000 1011 10.1111/j.1365-2702.2006.01760.x 17518876

[B23] New South Wales Government, Western Sydney Local Health District 2013 http://www.wslhd.health.nsw.gov.au/Home

[B24] McGrath P Patton M Holewa H Rayner R The importance of the ‘Family Meeting’ in health care communication with indigenous people: findings from an Australian study Aust J Prim Health 2006 12 1 56 64 10.1071/PY06009

[B25] DiGiacomo M Thompson S Smith J Taylor K Dimer L Ali M Wood M Leahy T Davidson P ‘I don’t know why they don’t come’: barriers to participation in cardiac rehabilitation Aust Health Rev 2010 34 452 457 10.1071/AH09803 21108907

[B26] Ritchie J Spencer L Bryman A, Burgess R Qualitative data analysis for applied policy research Analysing qualitative data 1993 London: Routledge 173 194

[B27] Thomas D A general inductive approach for analyzing qualitative evaluation data Am J Eval 2006 27 2 237 246 10.1177/1098214005283748

[B28] Thorne J Middle ear problems in Aboriginal school children cause developmental and educational concerns Contemp Nurse: A Journal for the Australian Nursing Profession 2003 16 1–2 145 150 10.5172/conu.16.1-2.145 14994905

[B29] DiGiacomo M Davidson PM Abbott P Delaney P Dharmendra T McGrath SJ Delaney J Vincent F Childhood disability in aboriginal and torres strait islander peoples: a literature review BMC Int J Equity in Health 2013 12 1 7 http://www.equityhealthj.com/content/pdf/1475-9276-12-7.pdf 10.1186/1475-9276-12-7 PMC3641946 23327694

[B30] O’Neill M Kirov E Thomson N A review of the literature on disability services for Aboriginal and Torres Strait Islander peoples Aust Indigenous Health Bull 2004 4 4 1 26

[B31] Gunasekera H Morris PS Daniels J Couzos S Craig JC Otitis media in Aboriginal children: The discordance between burden of illness and access to services in rural/remote and urban Australia J Paediatr Child Health 2009 45 7/8 425 430 19722295 10.1111/j.1440-1754.2009.01532.x

[B32] Burrow S Galloway A Weissofner N Review of educational and other approaches to hearing loss among Indigenous people Australian Indigenous HealthBulletin 2009 2 1 http://www.healthinfonet.ecu.edu.au/other-health-conditions/ear/reviews/our-review-education

[B33] Brough M National emergency response to protect children in the NT, Media Release Australian Government 2007 Canberra: Department of Families, Community Services and Indigenous Affairs

[B34] Aboriginal Disability Network NSW Aboriginal Disability Network NSW Conference Report First State Aboriginal Disability Network NSW conference: 18–20 November 2002; Gibba Gunyah Stone Quarry Lodge, Picton, NSW 2002 Redfern: Aboriginal Disability Network New South Wales 8

[B35] Walker K National preschool education inquiry report - ‘For all our children’: report of the independent inquiry into the provision of universal access to high quality preschool education 2004 Melbourne, Vic: Australian Education Union 74p

[B36] Vass AAY Health literacy and Australian Indigenous peoples: an analysis of the role of language and worldview Health Promot J Austr 2011 22 1 33 37 21717835 10.1071/he11033

[B37] Nelson A Allison H A visiting occupational therapy service to indigenous children in school: results of a pilot project Aust J Indigenous Educ 2004 33 2004 55 60

[B38] Nelson A Allison H Relationships: the key to effective occupational therapy practice with urban Australian Indigenous children Occup Ther Int 2007 14 1 57 70 10.1002/oti.224 17623379

[B39] Abbott P Gordon E Davison J Expanding roles of Aboriginal health workers in the primary care setting: seeking recognition Contemp Nurse: A Journal for the Australian Nursing Profession 2008 27 2 157 10.5172/conu.2008.27.2.157 18457516

[B40] National Aboriginal Community Controlled Health Organisation (NACCHO) and the Royal Australian Council of General Practicitioners (RACGP) National guide to a preventive health assessment for Aboriginal and Torres Strait Islander people 2012 2 South Melbourne: RACGP

[B41] Henderson I Remote area aboriginal ear and hearing health: who defines the problem? Aborig Isl Health Work J 1993 17 4 19 22

[B42] DeBats R Aboriginal students with disabilities 2003 Adelaide: Ministerial Advisory Committee

[B43] Heckman J Investing in disadvantaged young children is an economically efficient policy Committee for Economic Development 2006 New York: Pew Charitable Trusts

